# Seeing biomass recalcitrance through fluorescence

**DOI:** 10.1038/s41598-017-08740-1

**Published:** 2017-08-18

**Authors:** Thomas Auxenfans, Christine Terryn, Gabriel Paës

**Affiliations:** 1 FARE laboratory, INRA, University of Reims Champagne-Ardenne, 2 esplanade Roland-Garros, 51100 Reims, France; 2 PICT platform, University of Reims Champagne-Ardenne, 45 rue Cognacq-Jay, 51100 Reims, France

## Abstract

Lignocellulosic biomass is the only renewable carbon resource available in sufficient amount on Earth to go beyond the fossil-based carbon economy. Its transformation requires controlled breakdown of polymers into a set of molecules to make fuels, chemicals and materials. But biomass is a network of various inter-connected polymers which are very difficult to deconstruct optimally. In particular, saccharification potential of lignocellulosic biomass depends on several complex chemical and physical factors. For the first time, an easily measurable fluorescence properties of steam-exploded biomass samples from miscanthus, poplar and wheat straw was shown to be directly correlated to their saccharification potential. Fluorescence can thus be advantageously used as a predictive method of biomass saccharification. The loss in fluorescence occurring after the steam explosion pretreatment and increasing with pretreatment severity does not originate from the loss in lignin content, but rather from a decrease of the lignin β-aryl-ether linkage content. Fluorescence lifetime analysis demonstrates that monolignols making lignin become highly conjugated after steam explosion pretreatment. These results reveal that lignin chemical composition is a more important feature to consider than its content to understand and to predict biomass saccharification.

## Introduction

Lignocellulosic biomass is considered as a sustainable and alternative source of fuels, chemicals and materials. It refers to plant dry matter from various sources which are available or grown nearly in any location on Earth: agricultural by-products (e.g. cereal leaves and straws), wood (e.g. forest trees and short rotation crops) and dedicated crops (e.g. miscanthus). Lignocellulosic biomass production (crop residues and wood) in Europe was close to 750 million tons in 2011^1^. In comparison to another plant product like starch, lignocellulosic biomass does not compete with feedstock. Therefore, valorisation of lignocellulosic biomass is expected to favour the transition from a fossil carbon-based to a green carbon-based economy, thus limiting greenhouse gas emission and climate changes which are strong policy priorities in Europe^1^.

Lignocellulosic biomass from grass and wood is mainly composed of three types of polymers that account for more than 90% of their dry weight: cellulose, hemicellulose and lignin^2^. Such polysaccharides and polyphenols are of great interest for producing green chemicals and materials, for example to produce composites including fibres or to ferment sugars in different compounds, including biofuels. But the high chemical and structural complexity of lignocellulosic biomass at different length-scales is also a limitation for the development of economically viable transformation^3^: that is why lignocellulosic biomass is known as a recalcitrant material^4, 5^.

Different routes exist for processing lignocellulose, often involving some pretreatments which are due to increase the efficiency of catalysts leading to products of interest^6^. The use of enzymes and micro-organisms is relevant since they are more selective (enzymes are specific catalysts) and saves more energy (enzymes work in mild temperature conditions and can often be recycled). But enzymatic catalysts which perform the hydrolysis reaction are nearly inactive if they are applied directly onto native raw lignocellulose biomass. A physicochemical pretreatment is necessary, to make the substrate less recalcitrant. Only after, a specific enzymatic hydrolysis of cellulose and hemicellulose is performed, in order to obtain the highest rate of sugars subsequently fermented by yeasts into fuels and chemicals^7^.

Making lignocellulose biomass less recalcitrant goes through the development of optimal pretreatments to favour the action of enzymes and of cellulases in particular^8, 9^. Many different pretreatments have been studied and can be applied to lignocellulosic biomass^10^, each having some advantages and drawbacks regarding efficiency, cost and production of inhibitors^11^. One efficient and industrially well-established pretreatment is steam explosion (SE). During SE, chipped biomass is subjected to high pressure water steam at temperatures ranging from 170 to 220 °C for a few minutes before a rapid return to atmospheric pressure, resulting in the biomass explosion^10^. SE efficiency is mainly due to the large increase of pore size and accessible surface area, combined with hemicellulose hydrolysis and partial lignin solubilisation^10, 12, 13^. Some acid catalysts such as dilute sulfuric acid can also be added to increase SE efficiency. Temperature and time of SE process have to be carefully controlled according to biomass characteristics (species, particle size, moisture) since some hydrolysis and fermentation inhibitors can be generated under high severity conditions^11, 14^.

Saccharification of pretreated biomass is dependent on many different physical and chemical factors^15^, so predicting glucose yield based on easily measurable parameters is very challenging. Lignin content and cellulose crystallinity are generally recognized as dominating factors^16^. Accessible surface area of pine wood pretreated with ionic liquid was perfectly fitted with saccharification, contrarily to lignin content^17^. The role of water interactions (state and location) with lignocellulose was also shown to be important in SE biomass^18^. Recent in-depth statistical analysis of the weight of different structural factors controlling hydrolysability of pretreated biomasses showed that many factors were involved and not a single one could explain saccharification^19–21^. Moreover, measuring parameters such as lignin content, cellulose crystallinity or porosity (for example through Simons’s staining or thermoporosimetry) is not straightforward nor rapid. Spectral parameters from infra-red spectroscopy data can more advantageously be used to accurately predict glucose yield after hydrolysis of wheat straw^22, 23^. Overall, relative importance of chemical and structural factors depends on biomass species, pretreatment type and process conditions, no general factor has been revealed until now^24^.

Another experimental parameter that can be easily measured on lignocellulosic samples is fluorescence. Plant cell wall are autofluorescent materials, containing some endogenous fluorophores, in particular aromatic molecules: monolignols in lignin^25^, ferulic acid and cinnamic acids in hemicellulose^26^. Lignin content in biomass feedstock is in the range 15–30%^2^. It is made of three different monolignols: coniferyl alcohol (CA), *p*-coumaryl alcohol and sinapyl alcohol (SA)^25^. Interestingly, these monolignols are non-conjugated moieties but display a high fluorescence^27^. Lignin autofluorescence is typically multimodal^28^ and highly complex, as revealed for example through lifetime imaging indicating variable spatial lignin distribution and spectral properties^29^. For imaging purpose, lignin autofluorescence is detrimental for detecting transgenic proteins *in planta* but particularly useful for observing plant cell wall architecture^30^. Overall, cell wall fluorescence depends on lignin composition, type and content of inter-linkages between monolignols and surrounding environment (vicinity of other fluorophores, interactions with other molecules, pH, ionic strength). So all these parameters possibly affect fluorophore extinction coefficient, quantum yield and lifetime thus modifying the fluorescence absorption, emission and intensity^31^. Goal of this study is to explain the loss in fluorescence observed in steam-exploded biomass samples and how it can be directly correlated to biomass digestibility.

## Results

Enzymatic saccharification of biomass samples from miscanthus, poplar and wheat straw was conducted for 48 hrs and glucose concentration was evaluated for the untreated and pretreated samples at different severity factors (CSF) (Table 1 from ref. 32). Glucose release was lower in untreated samples, with notable difference between poplar (less than 1 g/L, corresponding to a glucose conversion yield of 7%) and wheat straw (more than 4 g/L, glucose yield of 51%). For samples pretreated with a severity of 2.0, glucose concentration reached 8–9 g/L and even more than 10 g/L for CSF 2.8, the highest severity tested. This means that pretreatment has increased by more than 2-fold (for wheat straw) to 10-fold (for poplar) the release of glucose, so that glucose yield was close to 100%. These results are in agreement with those previously published^33^.

**Table 1:** Glucose concentration in g/L after a 48-hour saccharification by the Cellic CTec2 cocktail. NA: sample not available.

	Miscanthus	Poplar	Wheat straw
Untreated	1.82 ± 0.09	0.71 ± 0.13	4.41 ± 0.06
Pretreated - CSF 2.0	8.42 ± 0.18	7.87 ± 0.66	9.06 ± 0.02
Pretreated - CSF 2.6	10.48 ± 0.38	9.87 ± 0.23	10.46 ± 0.02
Pretreated - CSF 2.7	10.67 ± 0.47	NA	10.31 ± 0.10
Pretreated - CSF 2.8	10.11 ± 0.43	10.15 ± 0.51	10.36 ± 0.39

Means and standard deviations are based on measurements done in triplicate.

**Figure 1:**
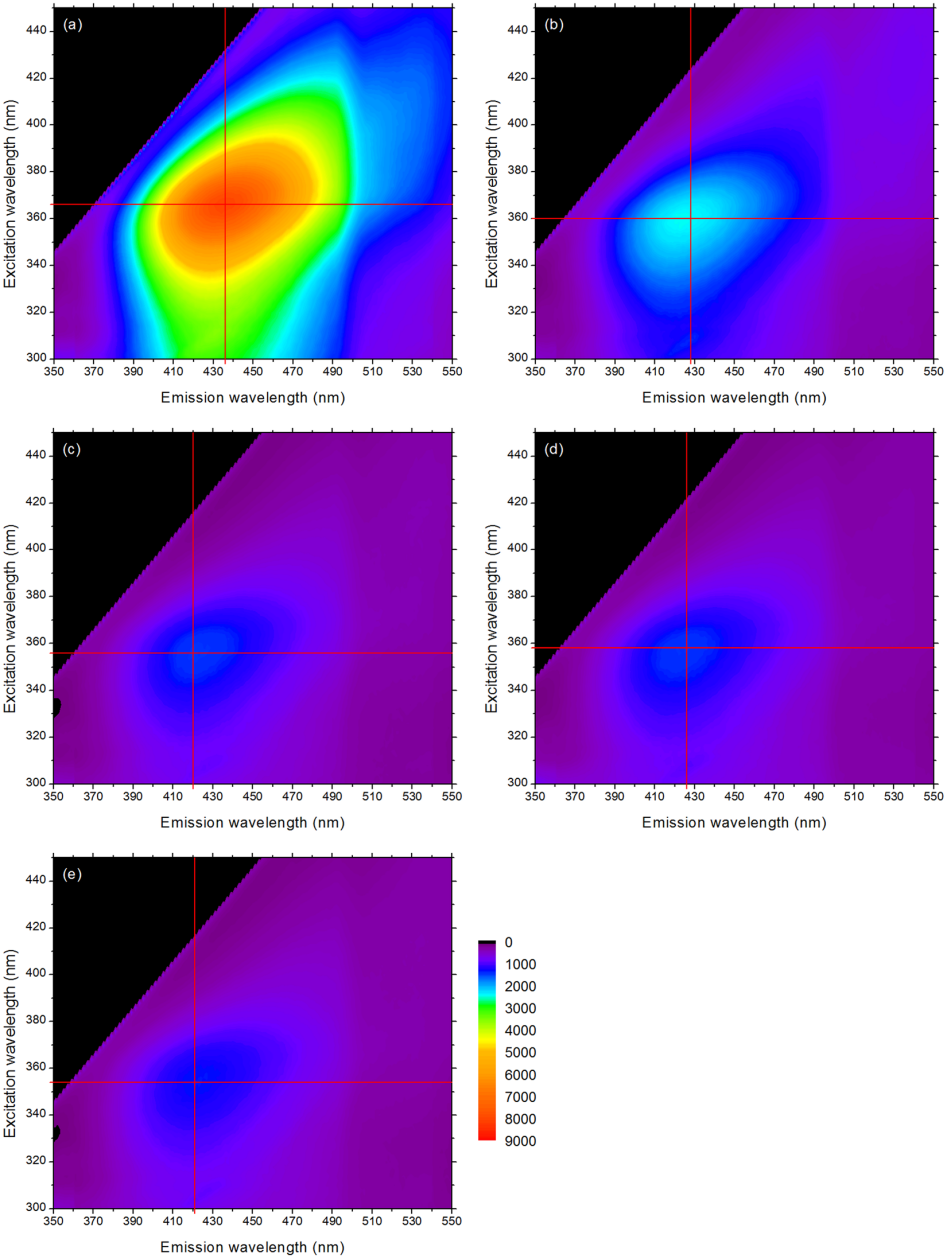
3D fluorescence contour map of miscanthus (**a**) untreated sample and pretreated samples with (**b**) CSF = 2.0, (**c**) CSF = 2.6, (**d**) CSF = 2.7, (**e**) CSF = 2.8. The red cross indicates the maximum fluorescence intensity value.

The same samples used for the saccharification measurements were used to prepare some KBr disks. KBr is commonly mixed with samples to be analysed by infrared spectroscopy since it is known to have a very low spectral absorption^34^. Some disks containing only KBr were prepared and analysed to check KBr absorption in the range 300–800 nm was negligible (data not shown). Fluorescence spectra measurements were performed for all untreated and pretreated samples with the same acquisition parameters. Only the gain value was adapted for each biomass species to get the highest fluorescence intensity value for the untreated samples. Fluorescence spectra are presented as 3D contour maps (Fig. 1; Supplementary Figs 1 and 2). For facilitating comparison, the maximum fluorescence intensity values were determined and are reported in Table 2, with corresponding maximum excitation and emission values. For miscanthus, untreated sample maximum fluorescence intensity (Fig. 1) reaches more than 8000. Even for the less drastically pretreated sample, maximum intensity is decreased by more than 3-fold. When CSF increases, intensity continues to decrease to be finally divided by 6. For poplar, untreated sample fluorescence intensity (Supplementary Fig. 1) also reaches more than 8000, but pretreated sample with CSF 2.0 has a maximum intensity only decreased by a bit more than 2-fold. Again, intensity is minimum for the most severely treated sample and gets divided by 6. Finally, for wheat straw (Supplementary Fig. 2), shape of the 3D map of the untreated sample is slightly different: instead of being circular like for miscanthus and poplar, it displays a larger surface for high excitation and emission values. Interestingly, fluorescence intensity for sample with CSF 2.0 is only decreased by 30%, and decreased by less than 50% for the most drastically treated sample.

**Table 2:** Maximum fluorescence intensity and corresponding excitation (λ_EX_) and emission (λ_EM_) wavelengths of untreated (UN) and pretreated samples from Fig. 1 and Supplementary Figs 1 and 2.

	Miscanthus			Poplar			Wheat straw		
	Maximum fluorescence intensity	λ_EX_ (nm)	λ_EM_ (nm)	Maximum fluorescence intensity	λ_EX_ (nm)	λ_EM_ (nm)	Maximum fluorescence intensity	λ_EX_ (nm)	λ_EM_ (nm)
UN	8100	366	436	8307	376	445	7536	358	441
CSF 2.0	2450	360	428	3225	358	416	5086	352	424
CSF 2.6	1480	356	420	1862	360	422	3921	348	422
CSF 2.7	1462	358	426	ND	ND	ND	4030	352	422
CSF 2.8	1342	354	421	1409	358	418	3994	352	417
Δ_CSF 2.8/UN_	−84%	−12	−15	−83%	−18	−27	−47%	−6	−24

Standard deviations from fluorescence intensity measurements done in triplicate are below 5%. Δ_CSF 2.8/UN_: variation between pretreated sample with CSF 2.8 and untreated sample.

Examination of maximum excitation and emission wavelengths (λ_EX_ and λ_EM_) variations depending on pretreatment severity (Table 2) shows important decreases of both parameters: λ_EX_ is shifted from −6 to −18 nm (hypsochromic effect) while λ_EM_ is more altered with variations from −15 to −27 nm. Overall, each biomass has a different fluorescence profile along pretreatment severity: miscanthus is the most affected for the intensity loss, while poplar seems a little less modified for intermediate severity samples but maximum λ_EX_ and λ_EM_ are largely altered; wheat straw appears as the sample for which fluorescence is much less affected by pretreatment. Considering samples analysed, pretreatment has led to important decreases of fluorescence intensity (hypochromic effect) and to a blue-shift of both λ_EX_ and λ_EM_ (hypsochromic effect).

**Figure 2:**
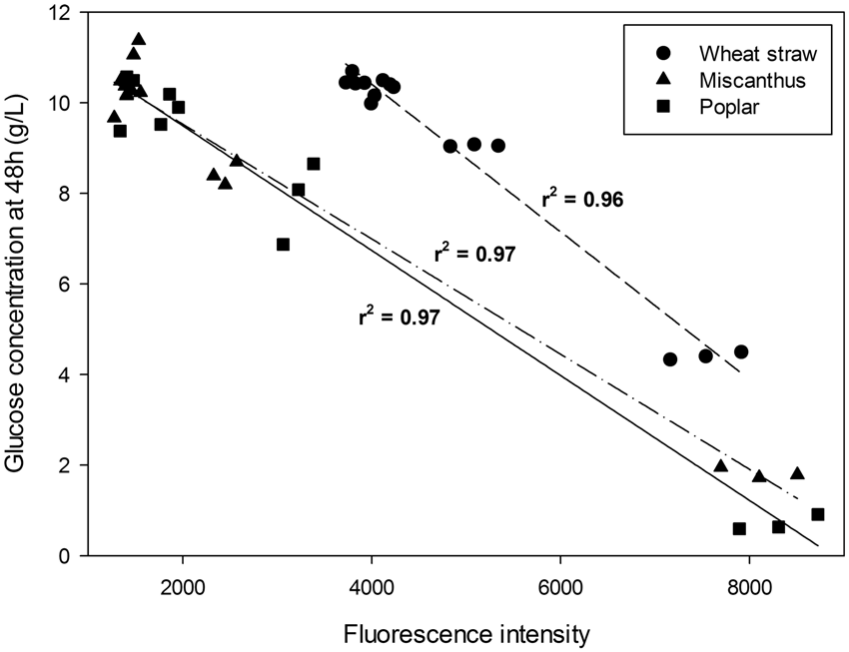
Correlation between glucose concentration released after 48 hrs saccharification (from Table 1) and maximum fluorescence intensity (from Table 2) of untreated and pretreated samples. Correlation coefficient and related correlation line are drawn for each biomass sample (wheat straw: short dash line; miscanthus: dash and dot line; poplar: solid line).

It has been shown previously on the same samples^32^ that when pretreatment severity was increased, saccharification was largely improved, while fluorescence intensity was reduced. Given that the fluorescence measurements can be easily carried out, it seemed interesting to try correlating the fluorescence intensity measured for each sample with the corresponding saccharification data. As a result, glucose concentration released after 48 hrs saccharification was plotted against maximum fluorescence intensity (Fig. 2). Calculated correlation coefficients indicated a very strong correlation between the two parameters since *r* ^2^ was in the range 0.96–0.97 (Fig. 2). Getting such a good agreement using between 12 and 15 different analysed samples for each biomass is particularly strong. To our knowledge, this is the first time that the glucose concentration released after the saccharification of untreated and steam-exploded samples from three different biomass species and using a commercial enzyme cocktail can be simply described by fluorescence measurement.

## Discussion

**Figure 3:**
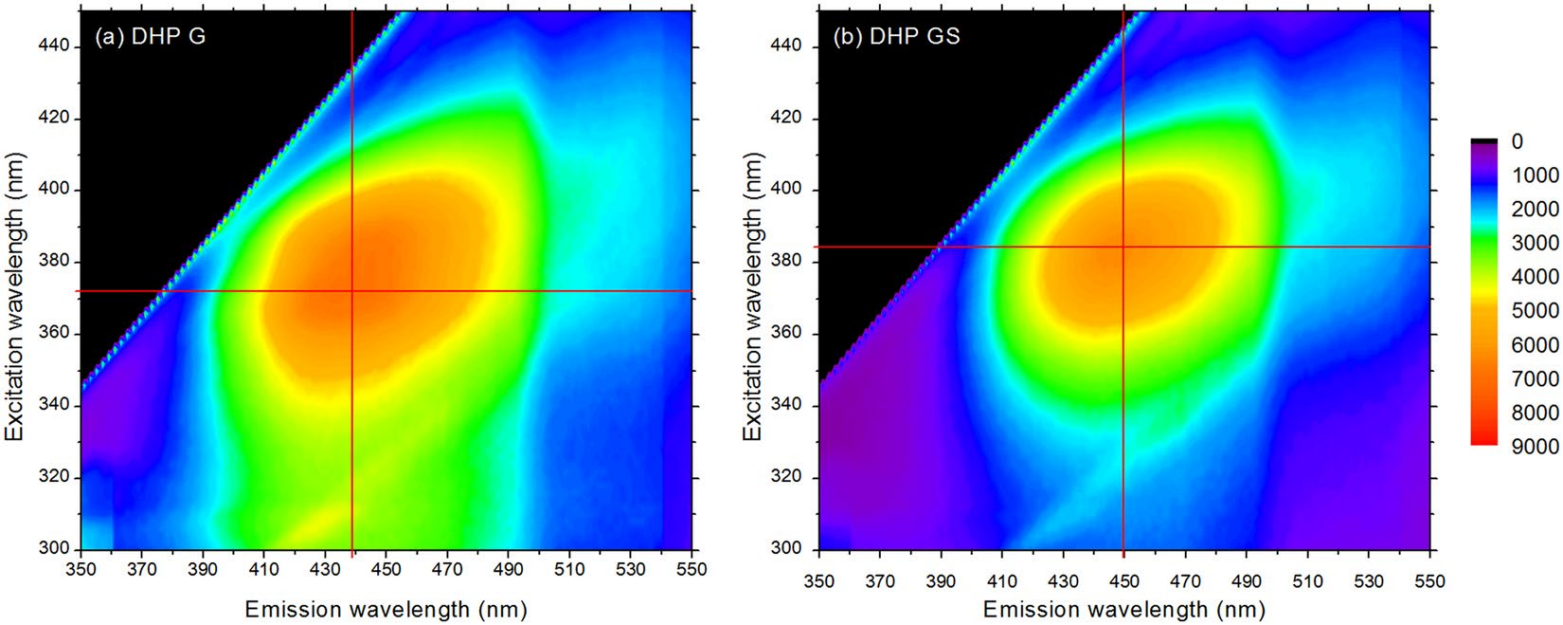
3D fluorescence contour map of model lignin DHPs: (**a**) DHP G made of 100% coniferyl alcohol and (**b**) DHP GS made of 50% coniferyl alcohol +50% sinapyl alcohol. The red cross indicates the maximum fluorescence intensity value.

Effect of SE pretreatment on plant cell walls from different species has been well characterized regarding accessibility, chemical modifications and saccharification^11, 20^. But few studies have analysed the induced modification of lignin and its fluorescence. More generally, origin of lignin fluorescence has been mostly investigated in model lignin compounds^35^. In-depth characterization of model CA dilignols suggested that fluorophores are not significantly electronically coupled to one another and can be seen as isolated fluorophores^27^. But even model lignin polymers fluorescence differs significantly depending on their composition^36^. Recently, it has been proposed that lignin fluorescence may originate from its aggregation due in particular to clustering of the carbonyl groups^37^. Since the origin of lignocellulose fluorescence is questionable, some model lignin dehydrogenative polymers (DHPs) made of 100% CA (DHP G) and 50% CA + 50% SA (DHP GS) were synthesized^38^. Their fluorescence spectra (Fig. 3) indicates maximum λ_EX_/λ_EM_ of 372 nm/439 nm and 382 nm/450 nm, respectively, in agreement with the values previously presented^36^. Patterns of native lignocellulosic samples (Fig. 1, Supplementary Figs 1 and 2) and of DHPs (Fig. 3) are very similar, demonstrating that autofluorescence of biomass mainly originates from lignin fluorescence.

Based on these information, the important loss in fluorescence observed in the SE samples can be attributed to one or several of the following causes: decrease of lignin content; alteration of linkages between monolignols making lignin; modification of the cross-linkages between lignin and hemicellulose and of the organization of the polymers (mainly cellulose and hemicellulose) surrounding lignin. If SE samples fluorescence was expected to be mainly influenced by the packing polymers around lignin, a possible approach could be to assay the lignin chemical and physical accessibility in the pretreated samples^39, 40^, but since samples were “exploded” after pretreatment, these techniques could not be applied. Previously, an in-depth chemical analysis of the same pretreated samples was performed. Results have shown that lignin content increased with increasing severity^32^ (Table 3) due to hemicellulose hydrolysis^10^. So loss in fluorescence cannot be attributed to some variations in the content of lignin.

**Table 3:** Content in lignin and β-O-4′ inter-unit cross-linkages of untreated (UN) and pretreated samples (from ref. 32) and correlation coefficient between [β-O-4′] and fluorescence maximum intensity (from Table 2).

		[lignin] (% in weight)	[β-O-4′] (% of aromatic rings)	Fluorescence maximum intensity	Correlation coefficient
Miscanthus	UN	25.5	42.1	8100	0.99
	CSF 2.0	27.2	13.3	2450	
	CSF 2.6	29.1	8.5	1480	
	CSF 2.7	29.6	4.2	1462	
	CSF 2.8	29.7	ND	1342	
Poplar	UN	29.5	43.1	8307	0.91
	CSF 2.0	30.5	31.0	3225	
	CSF 2.6	33.0	13.0	1862	
	CSF 2.7	ND	ND	ND	
	CSF 2.8	33.4	6.8	1409	
Wheat straw	UN	24.6	45.3	7536	0.99
	CSF 2.0	24.6	20.3	5086	
	CSF 2.6	27.3	12.4	3921	
	CSF 2.7	27.2	8.1	4030	
	CSF 2.8	30.1	9.3	3994	

**Figure 4:**
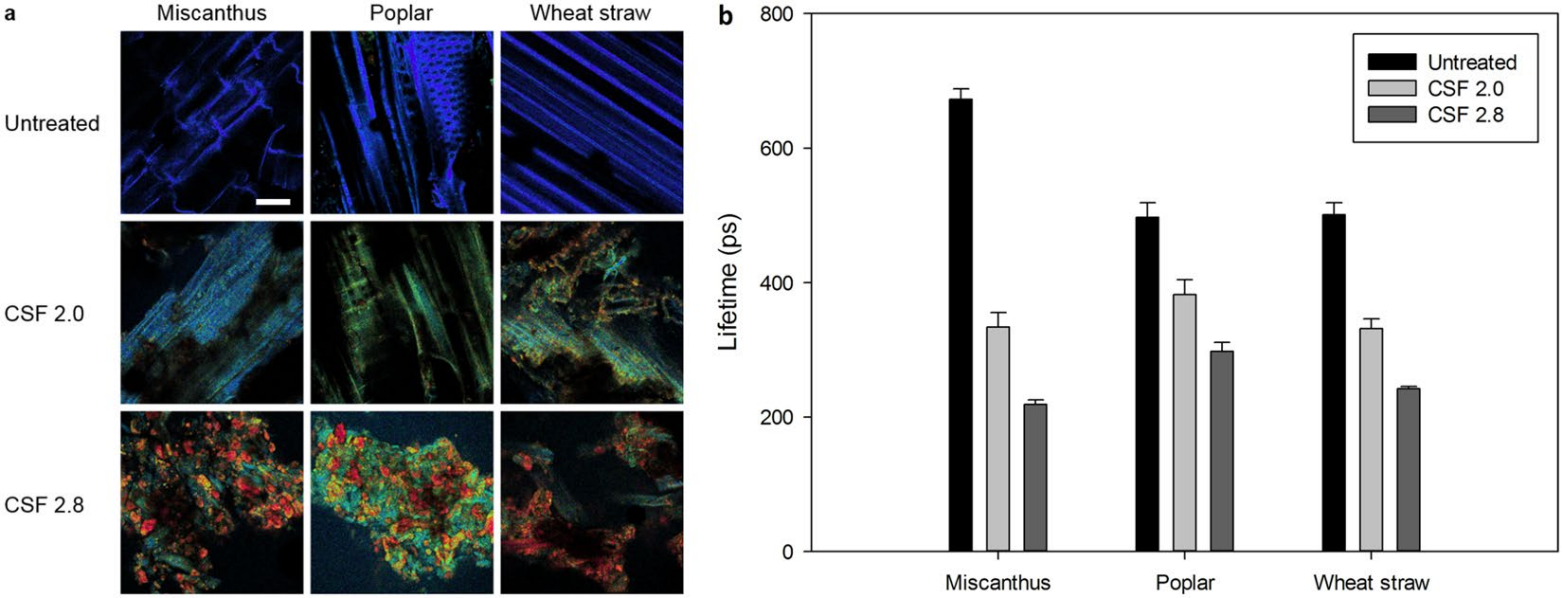
Fluorescence properties of untreated and pretreatd samples. (**a**) Spectral image of autofluorescence after a bi-photon excitation at 750 nm, emission measured from 420 nm (blue) to 722 nm (red); scale-bar is 10 µm. (**b**) Fluorescence lifetime of untreated and pretreated samples. Fluorescence lifetime values are averaged from measurements on three different samples.

Besides, content in lignin inter-unit linkages (β-O-4′, β-5′, and β-β′) was also determined by 2D NMR^32^ (Table 3). In particular, the β-aryl ether linkages (β-O-4′) content was noticed to decrease with increasing pretreatment severity. Determination of the correlation coefficient between the β-aryl ether linkage content and the fluorescence maximum intensity for each untreated and pretreated biomass sample gives a very strong positive correlation (Table 3). If this correlation seems rational based on the fact that fluorescence of plant cell wall originates from lignin, this means that other parameters characterizing fluorescence, namely maximum fluorescence emission intensity and fluorescence lifetime (τ), should also be affected by SE pretreatment^31^. First, to illustrate the impact of pretreatment on fluorescence emission, spectral imaging of SE samples was done with a 750 nm biphoton wavelength (Fig. 4a). No differences based on biomass species could be noticed, rather an increasing shift of fluorescence emission with increasing severity was observed. Fluorescence lifetime of a fluorophore describes the fluorescence decay from an excited state to the ground state. Dissipation of energy through the emission of a photon is notably favoured by the presence of electron-rich bonds^41^. Determination of fluorescence lifetime of SE samples shows that untreated samples have higher τ values than pretreated samples, whatever the biomass species considered are (Fig. 4b). This means that high severity pretreatment, which was shown to be accompanied by lignin recondensation^32^, modifies the electronic environment of lignin, probably by the creation of highly conjugated monolignols. Overall, results on fluorescence characterization show that the loss in fluorescence observed after SE pretreatment can be directly attributed to the degradation of the β-aryl-ether linkages occurring during lignin depolymerisation/recondensation. In addition, recondensed lignin becomes an electron-dense polymer, despite the loss of β-aryl-ether linkages, involving the creation of uncharacterized linkages.

Regarding the correlation between biomass autofluorescence and saccharification potential, it was previously tested by measuring fluorescence, but at only two excitation wavelengths (405 and 561 nm), and only qualitative trends could be proposed^42^. Contrarily to this study and others^43^, biomass autofluorescence analysis presented here has been thoroughly made on a wide range of excitation wavelengths (300–450 nm), which has led to uncovering this new correlation between sample fluorescence and saccharification rate. So even if lignin content and saccharification efficiency are considered to be negatively correlated^15^, it does not seem to be a general trend. Rather, it seems pretreatment type and characterization of pretreatment effect on lignin chemical properties are more important to consider than lignin content alone to explain saccharification efficiency^44^. This result is for example consistent with the higher saccharification measured in transgenic poplar in which β-aryl-ether linkage content was decreased^45^. Consequently, this parameter is particularly important to take into account in the frame of pretreatment conditions optimisation and in the design of genetically modified lignins^45–47^.

In summary, pretreatment of lignocellulosic biomass is a crucial step in biorefineries to increase the accessibility of enzymes and to optimize their activity. SE is recognized as one of the most efficient existing pretreatments. But depending on biomass origin, the different parameters controlling the steam explosion step (temperature, pH, acid content) must be adapted. Consequently, biomass is more or less affected regarding its architecture and composition, so that saccharification can liberate more or less glucose. The simple, fast and cheap measurement of 3D fluorescence contour maps has been shown to be highly correlated to the glucose concentration released after 48 hrs. This technique can thus be used as a predictive method to determine the saccharification rate of steam-exploded pretreated samples, saving much time and materials. The origin of the fluorescence loss after SE pretreatment was unambiguously attributed to the loss in β-aryl-ether linkages in lignin, likely accompanied by the creation of highly conjugated linkages between monolignols which remain to be characterized.

## Methods

### Biomass pretreatment and hydrolysis

Three native and steam exploded *Miscanthus* x *giganteus*, poplar and wheat straw residues were selected and provided by Procethol 2 G (Pomacle, France)^32^. Each experimental condition imparted different severity which can be expressed as a combined severity factor (CSF). CSF was calculated using the following equation^48^:

1 $$CSF={\mathrm{log}}_{10}(t\,\ast \,{\exp }^{\frac{(T-{T}_{R})}{14.75}})-pH$$

where *t* is the reaction time (min), *T* is the operating temperature (°C), *T*_*R*_ is the reference temperature (100 °C) and *pH* is that of the sulfuric acid solution used for biomass samples pre-soaking. The different CSF values ranged from 2.0 to 2.8.

Enzymatic saccharification assays of both native and steam exploded samples were used as received, without washing or further milling, using the commercial cellulase preparation Cellic CTec2 ®, courteously provided by Novozymes (Bagsværd, Danemark) as detailed previously^32^.

### Spectrofluorimetry

For other experiments, samples were ball-milled in a 25 mL jar with 20 × 20 mm ZrO_2_ ball bearings using a Retsch MM2000 mixer mill for 2 min to achieve a size less than 80 µm. Disks were prepared by mixing 2 mg of biomass sample and 200 mg of KBr. Fluorescence spectra of the disks were measured in a Jasco FP-8300 instrument (Lisses, France). Acquisition parameters were as follows: range/precision for excitation and emission were 250–600 nm/2 nm and 260–650 nm/1 nm, respectively; excitation and emission bandwidth was 2.5 nm; scan speed was 1000 nm/min; gain value was adapted depending on the biomass considered: 600 V, 650 V and 700 V for miscanthus, poplar and wheat straw, respectively. Spectra acquisition was performed using Jasco Spectra Manager software.

Dehydrogenative polymer (DHP) synthesis was performed as previously explained^38^ and their fluorescence spectra was recorded with the same parameters as above, using a gain value of 750 V.

### Autofluorescence and lifetime measurements

Spectral images were acquired using laser scanning microscope LSM 710 NLO Zeiss (Zeiss SAS, Germany) coupled with Chameleon TiSa accordable 80 Mhz pulsed laser (COHERENT,USA). Sample excitation was performed at 750 nm with two-photon laser and spectral images were acquired using spectral detector of the microscope on 32 channels between 420 and 722 nm with 20× objective (NA 0.8).

Lifetime measurements were acquired using a MW-FLIM detector system along the SPC 150 photocounting card from Becker & Hickl (Becker & Hickl, Germany). This system allowed to do lifetime measurements along 12.5 ns time-windows with a 16 spectral channels detector driven by SPCM software (Becker & Hickl). Lifetime trace of emitted photons was accumulated during 30 s simultaneously on all spectral channels of the MW-FLIM detector. Each lifetime trace was acquired on 1024 temporal channels. Lifetime traces were then processed using SPCMImage software (Becker & Hickl).

## Electronic supplementary material

Supplementary Figures
