# Supplementary material for: Seeing biomass recalcitrance through fluorescence
Source: Sci Rep. 2017 Aug 18;7:8838. doi: 10.1038/s41598-017-08740-1 (PMC5562871; doi:10.1038/s41598-017-08740-1)
Supplement: Supplementary file 1 — Supplementary Figures [file 41598_2017_8740_MOESM1_ESM.pdf]

Seeing biomass recalcitrance through fluorescence

Thomas Auxenfans[1], Christine Terryn[2], Gabriel Paës[1]*

[1]FARE laboratory, INRA, University of Reims Champagne-Ardenne, 2 esplanade Roland-Garros, 51100 Reims, France

[2]PICT platform, University of Reims Champagne-Ardenne, 45 rue Cognacq-Jay, 51100 Reims, France

*Corresponding author: gabriel.paes@inra.fr

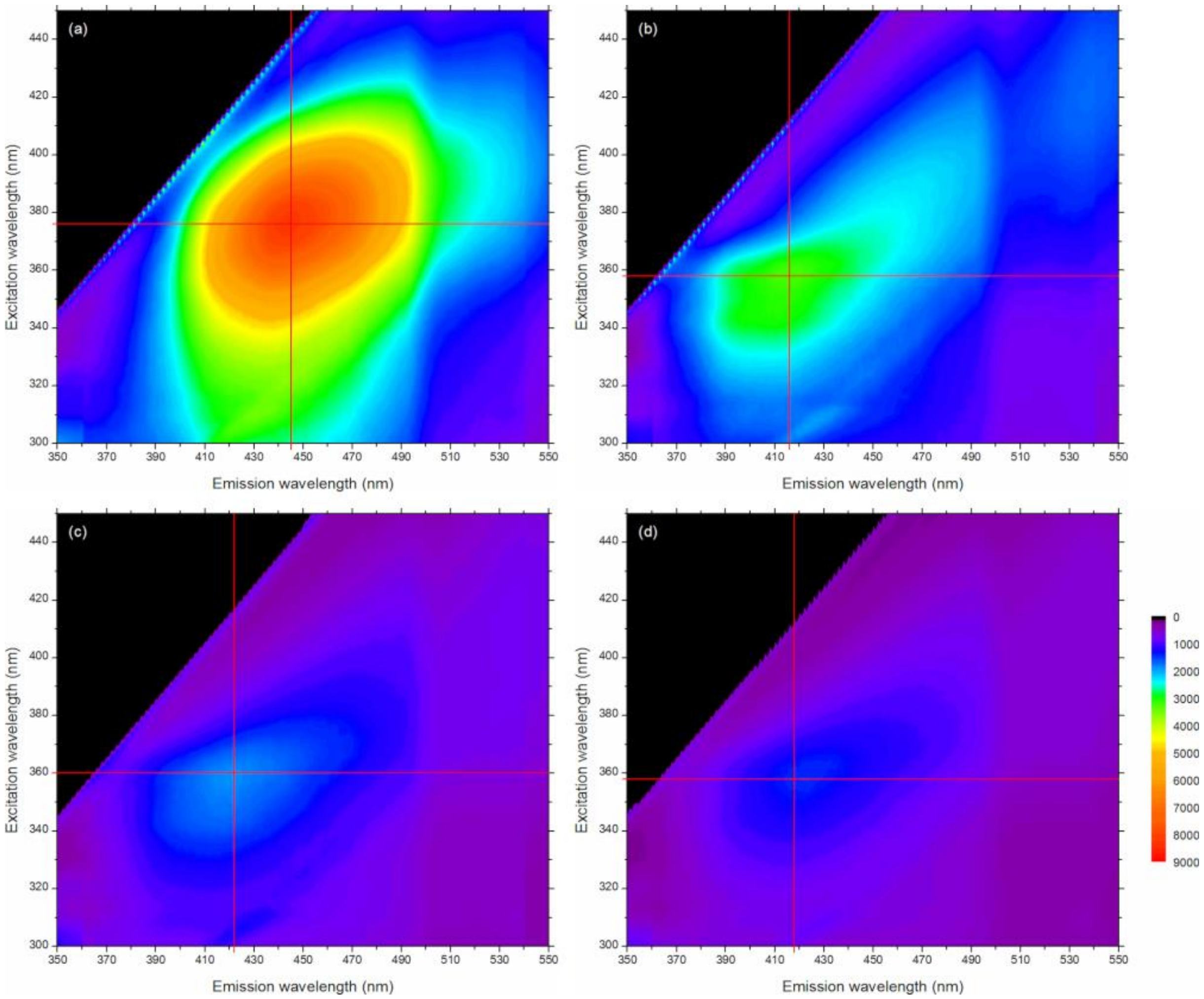

Supplementary figure 1. 3D contour map of poplar (a) untreated sample and pretreated samples with (b) CSF = 2.0, (c) CSF = 2.6, (d) CSF = 2.7, (e) CSF = 2.8. Fluorescence intensity is in arbitrary units. The red cross indicates the maximum fluorescence intensity value.

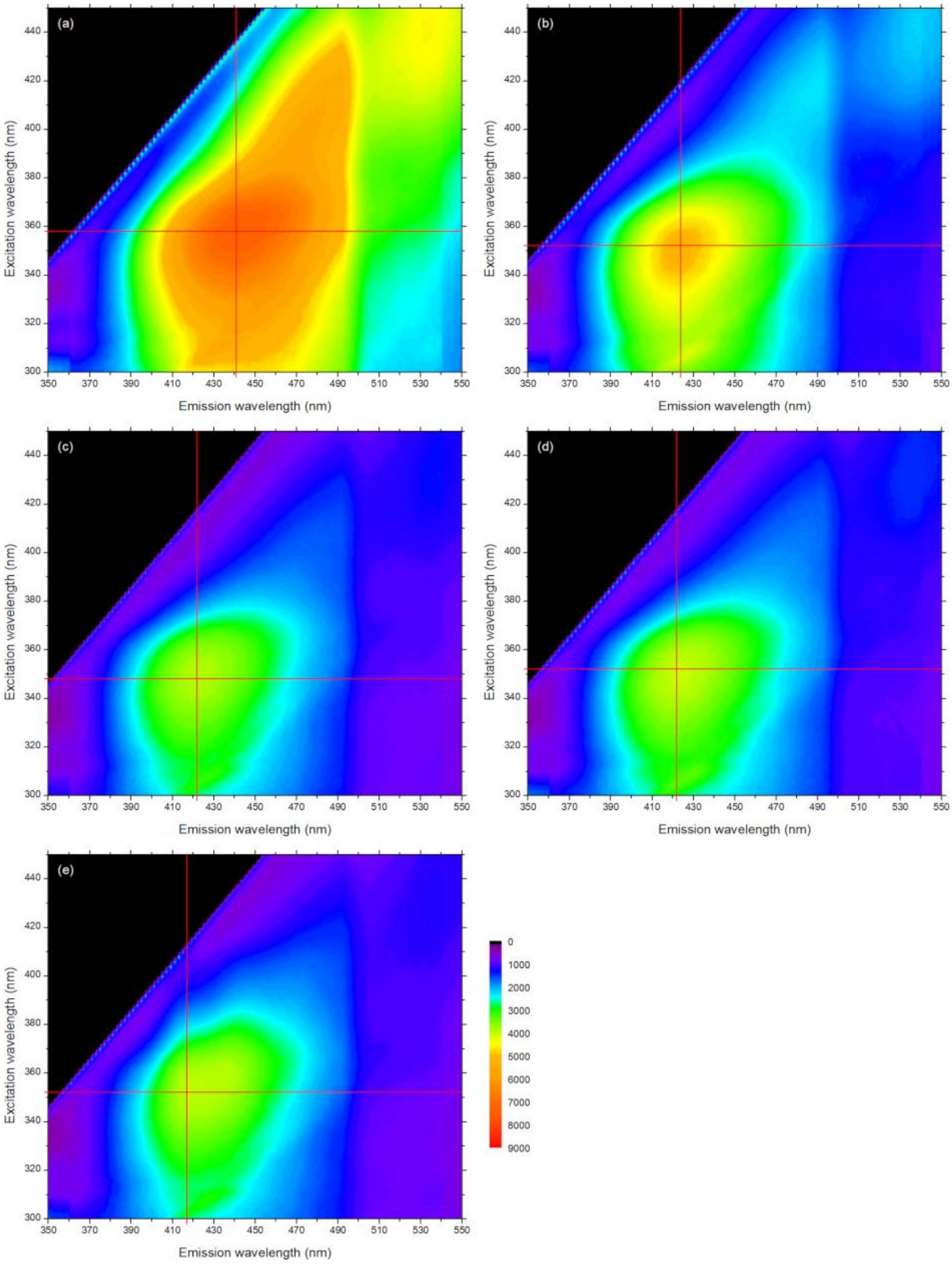

Supplementary figure 2. 3D contour map of wheat straw (a) untreated sample and pretreated samples with (b) CSF = 2.0, (c) CSF = 2.6, (d) CSF = 2.8. The red cross indicates the maximum fluorescence intensity value.
